# Obesity and type 2 diabetes mellitus: connections in epidemiology, pathogenesis, and treatments

**DOI:** 10.3389/fendo.2023.1161521

**Published:** 2023-04-21

**Authors:** Rexiati Ruze, Tiantong Liu, Xi Zou, Jianlu Song, Yuan Chen, Ruiyuan Xu, Xinpeng Yin, Qiang Xu

**Affiliations:** ^1^ Department of General Surgery, Peking Union Medical College Hospital, Chinese Academy of Medical Sciences and Peking Union Medical College, Beijing, China; ^2^ Key Laboratory of Research in Pancreatic Tumor, Chinese Academy of Medical Sciences, Beijing, China; ^3^ Graduate School, Chinese Academy of Medical Sciences and Peking Union Medical College, Beijing, China; ^4^ School of Medicine, Tsinghua University, Beijing, China

**Keywords:** obesity, diabetes mellitus, pathogenesis, islet function, microenvironment, bariatric surgery, β-cell failure

## Abstract

The prevalence of obesity and diabetes mellitus (DM) has been consistently increasing worldwide. Sharing powerful genetic and environmental features in their pathogenesis, obesity amplifies the impact of genetic susceptibility and environmental factors on DM. The ectopic expansion of adipose tissue and excessive accumulation of certain nutrients and metabolites sabotage the metabolic balance via insulin resistance, dysfunctional autophagy, and microbiome-gut-brain axis, further exacerbating the dysregulation of immunometabolism through low-grade systemic inflammation, leading to an accelerated loss of functional β-cells and gradual elevation of blood glucose. Given these intricate connections, most available treatments of obesity and type 2 DM (T2DM) have a mutual effect on each other. For example, anti-obesity drugs can be anti-diabetic to some extent, and some anti-diabetic medicines, in contrast, have been shown to increase body weight, such as insulin. Meanwhile, surgical procedures, especially bariatric surgery, are more effective for both obesity and T2DM. Besides guaranteeing the availability and accessibility of all the available diagnostic and therapeutic tools, more clinical and experimental investigations on the pathogenesis of these two diseases are warranted to improve the efficacy and safety of the available and newly developed treatments.

## 1 Introduction

The rapid development of modernization, urbanization, and accelerated socio-economic growth favored an improved living standard but a more stressful and sedentary lifestyle and unhealthy dieting habits in most parts of the world. Especially in the last two decades, obesity has become a global pandemic threatening people’s life by affecting almost every organ system and is now a severe public health problem as one of the most common non-communicable diseases (NCDs) (1–3). With no exception, all countries are now being affected by obesity, and this impact is predicted to be even more prominent during the current decade, resulting in more lost years of a healthy life, disability, and death (4). Hence, it is urgent to make effective and decisive actions to hinder the rise in the prevalence of obesity to prevent and treat obesity and other obesity-related comorbidities, among which T2DM is a health issue growing at an alarming speed in all regions as another global health emergency of the 21st-century (5). The booming increase in the prevalence of obesity in all age groups is one of the main culprits of the exponential growth of the population of T2DM (5).

Inspired by their connected epidemiology and plenty of clinical findings (6), researchers have made considerable efforts to investigate the deeper correlations between the pathogeneses of these two common metabolic disorders. The first to mention is the overlaps in their genetics/epigenetics revealed by the Genome-Wide Association Study (GWAS) (7), which are further dissected by multiomics technologies and other analytical approaches facilitating the translation of these genetic connections into shared etiological and pathophysiological features. Once obesogenic and diabetogenic environmental factors amplify the genetic susceptibilities, the ectopic expansion of adipose tissue and excessive accumulation of certain nutrients and metabolites sabotage the metabolic balance via insulin resistance, dysfunctional autophagy, and microbiome-gut-brain axis, which further exacerbate the dysregulation of immunometabolism through systemic inflammation (8), leading to an accelerated loss of β-cell function and gradual elevation of blood glucose.

Given these intricate connections in their pathogenesis, the current therapeutic options for managing and treating obesity and T2DM have partial similarities, ranging from lifestyle interventions, pharmacotherapy, and various newly developed medical devices and bariatric surgeries with increasing popularity and advancing techniques. Lifestyle interventions are the cornerstone and the frontline treatment method for obesity and T2DM, effective management of obesity is of top priority (9). While the anti-obesity drugs are anti-diabetic to some extent, some anti-diabetic medicines, however, have been shown to increase body weight, such as insulin. Meanwhile, taken for the backbone and the most effective treatment for both obesity and T2DM (10), surgical procedures, whether endoscopic or non-endoscopic, have made their names among patients suitable for different approaches, yet the inevitable risks and the complications still remind us that these techniques are far from perfection.

Although some connections between obesity and T2DM have been covered from different aspects in previous literature, their epidemiological, pathogenic, and therapeutic effects on each other haven’t been comprehensively described. Therefore, to provide a bigger picture of these existing tight bonds, the latest epidemiological data on obesity and T2DM will be parsed first to favor an overview of their linked prevalence. Then the main mechanisms responsible for their connections will be introduced, followed by detailed comparisons of the mutual effects of the available treatments for obesity and T2DM.

## 2 Epidemiological links between obesity and T2DM

### 2.1 Obesity

Determined by genetics, biology, healthcare availability, mental status, sociocultural factors, socioeconomics, personal lifestyle, and other environmental inducers, obesity is now generally recognized as a chronic, relapsing, and multifactorial disease dilatorily jeopardizing almost every organ system with its concurrent metabolic disorders or other related comorbidities such as T2DM, cardio- and cerebrovascular diseases, and cancers, impacting both physical and mental health in several ways that cannot be easily solved via weight loss (4). Usually, obesity is described by the body mass index (BMI). Still, this parameter suits more on the population level rather than defining the complexity of obesity as a disease, which requires a more comprehensive and systemic assessment (4).

In most developed countries, such as parts of Europe and North America, the rising prevalence of obesity is mainly caused by the growing socioeconomy, yet in many high-income and middle-income countries, the booming prevalence of obesity is fueled by the failure of obesity-tackling policies and inadequate comprehensive prevention, treatment, and management, with the discrepancies in the perception of obesity among different cultures also being important determinants (4). Currently, the prevalence of obesity in all age groups of both sex is booming around the world, with none achieving a decline in the obesity epidemic across its population, even though the global landscape differs among nations and the prevalence of obesity is plateauing at high levels in some regions, such as parts of Europe and North America (4, 11). The highest rates and numbers of people with obesity live in the World Health Organization (WHO) Americas region, yet the fastest growth in the population of obesity is now seen in low- and middle-income countries (LMICs) and small island developing states (SIDS) (4). Influenced by age and reproductive status (menopause etc.), the global prevalence of obesity is higher amongst women than men. According to the latest estimates, nearly 14% of men and 20% of women of the world’s population (over 1 billion people) will suffer from obesity by 2030 and the percentage of adults with obesity (Class I, II and III, BMI≥30kg/m^2^), severe obesity (Class II and III, BMI≥35kg/m^2^), and severe obesity (Class III, BMI ≥40kg/m^2^) will be 18%, 6%, and 2%, respectively (4). Regarding childhood obesity, the Western Pacific region has the highest prevalence and numbers, and the Middle East and Western Pacific regions are predicted to see a doubled population with childhood obesity by 2030, with the change being more attributable to the older age group (4).

The impacts and consequences of obesity are far beyond measure, which requires all countries to act on time with adequate efficiency. Still, the economic discrepancies are the root of the gaps in the capacity of obesity prevention amongst nations. According to the latest estimates of the World Obesity Federation (World Obesity), the LMICs are most unprepared, while the countries with higher income have a bigger capacity to embrace the challenge of obesity prevalence (4). Although action calls on obesity prevention have been being made since the 1970s (2), we haven’t done an excellent job in handling this one of the greatest threats to public health in the 21^st^ century, our latest failure of meeting the 2025 target to impede obesity prevalence early in 2020 (12) is already sending an alarm that the actual figure of the population with obesity in 2030 could perhaps be more unexpecting and astonishing if we don’t put more efforts into the global addressing of obesity. Of note, countries with a higher prevalence of obesity in adults but relatively lower in children stand a chance of benefitting more from powerful strategies of obesity prevention (4).

### 2.2 T2DM

The booming population of obesity around the globe is inevitably contributing to the increase in the prevalence of T2DM, which is also a chronic and overgrowing disease occurring when the body fails to produce enough (or any) insulin or cannot effectively use the insulin it produced, inducing elevated blood glucose (hyperglycemia) as a primary manifestation, ranking as one of the fastest-growing global health emergencies of this century (5).

Regardless of its types, the rapid increase in the prevalence of obesity is partially responsible for the equivalent rise in DM, affecting almost 10.5% of the world’s population, with the incidence of DM among youths also significantly increasing (5, 13, 14). According to the latest estimates of the International Diabetes Federation (IDF), almost half of the patients (240 million) with DM (mainly T2DM) are unaware of their condition and therefore undiagnosed, while the diagnosed DM was 537 million in 2021, which was predicted to be 643 million and 783 million by 2030 and 2045, respectively (5). When 90% of undiagnosed DM cases live in LMICs, and more than half of the patients with DM are undiagnosed in Africa, South-East Asia, and the Western Pacific, the incidence of DM was reported to be stable or declined in over 70% of mainly high-income populations during 2006 to 2017, same for more than 80% of countries since 2010 (5). Even so, there were other 541 million estimated to be prediabetic in 2021 (5). As more than 6.7 million death in the population aged 20~79 (equals12.2% of global deaths from all causes in this age group) was estimated to be caused by DM in 2021, the number of children and adolescents (under 19 years old) with DM is also increasing, of whom 1.2 million were diagnosed with type 1 DM (T1DM), and half of them are under 15 years old (5).

Being strongly connected to overweight and obesity, aging, ethnicity, and family history, T2DM accounts for more than 90% of DM worldwide, fueled by relative insulin deficiency owing to pancreatic β-cell dysfunction and insulin resistance (5). T2DM is preventable and manageable through education, support, lifestyle modification, required medication, and other available treatments, with accumulating evidence supporting the possibility of its remission as well (5). However, the insurmountable uncertainty of the exact time of the onset and the distinct duration of the prediabetic period leave nearly 30~50% of patients undiagnosed; thus, many patients are not diagnosed until treatments are required for the complications of T2DM (5). Set aside the massive population with currently diagnosed T2DM, countless people are at a high risk of developing future T2DM resulting from impaired glucose tolerance (IGT) and impaired fasting glucose (IFG), although the later onset of T2DM is likely to be preventable. In 2021, 541 million and 319 million adults were anticipated to have IGT and IFG, and these numbers were predicted to reach 730 million and 441 million by 2045 (5).

Obesity is also a solid influential driving factor of T2DM, which resulted in a nearly equivalent rise in T2DM epidemiology mainly through the adaptation of unhealthy diets, lack of physical activity, maternal obesity, etc. (5). Furthermore, growing numbers of older children and young people are also being diagnosed with T2DM due to the prevalence of childhood obesity. Compared to T1DM, youth with T2DM have a greater chance of developing cardiometabolic complications because of the elevated risk of hypertension, hyperlipidemia, and central obesity (5). However, the prevalence of childhood obesity is not definitely relevant to a higher incidence rate of youth-onset T2DM, and the prevalence of DM is higher in men than women in most of the age groups, which is quite the opposite of the higher prevalence of obesity amongst women than men (5), highlighting the substantial impact of other determinants and indicating that obesity is a potent booster of DM onset instead of being a decisive factor. Next, the main mechanisms connecting obesity and T2DM will be introduced in detail.

## 3 Mechanisms connecting obesity and T2DM

The consequence of obesity may seem quite straightforward—excessive weight gain. However, slowly increasing body weight is the fuse of subsequent metabolic disorders, among which T2DM is undoubtedly the one that is closely related to obesity. The outcome of metabolism-related T2DM is quite simple: hyperglycemia resulting from declined insulin sensitivity owing to the reduction of functional β-cell mass, with obesity being a powerful driver in its development and progression, including strengthened genetic/epigenetic vulnerability, microenvironmental changes impairing insulin signaling, deteriorated β-cell function, and dysregulated microbiome-gut-brain axis. However, T2DM can also occur inversely before obesity in some individuals with inherent insulin resistance resulting in increased hepatic glucose production and elevated insulin levels, which are the actual cause of obesity. This rare and challenging concept beyond the scope of this article is reviewed elsewhere (15), and the other commonly accepted mechanisms will be discussed below.

### 3.1 Genetics and environment affecting islet function

**Figure 1:**
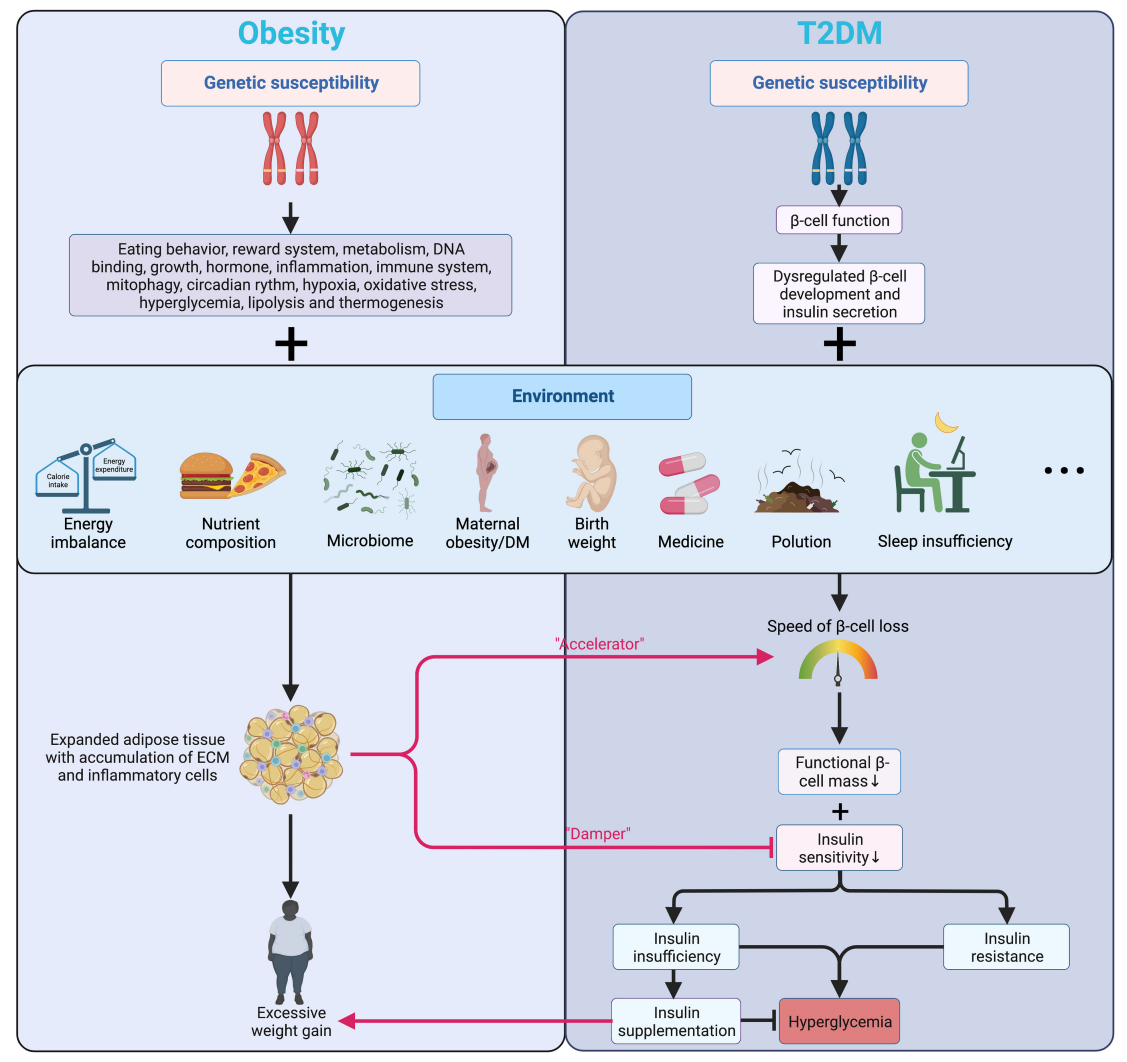
Genetic and environmental factors affecting islet function and connecting obesity and T2DM. Genetic factors mainly alter the energy balance in obesity while regulating the development and function of β-cells in T2DM. Being further promoted by various environmental factors, obesity accelerates the β-cell loss and blunts insulin signaling in T2DM. Meanwhile, insulin prescribed to patients with T2DM can have a weight-increasing effect. Arrows in color indicate the interactions between obesity and T2DM. T2DM, type 2 diabetes mellitus; ECM, extracellular matrix.

The pathogenesis of T2DM is characterized by the inflammatory component inducing progressive loss of β-cell insulin secretion with co-existing insulin resistance (16, 17), impacting early β-cell function and cell fate (18), where overweight and obesity are deemed the most effective “accelerator” (19) (**Figure 1**).

Many patients with obesity can go through a transitional stage called “Prediabetes” before eventually developing hyperglycemia, which refers to the scenario when the glucose levels are not high enough for a T2DM diagnosis while the normal carbohydrate metabolism is compromised (20). Despite not being a clinical entity but rather an omen prompting possible intervention and other comprehensive screening for T2DM and cardiovascular risk factors, prediabetes has a solid link to obesity (particularly abdominal or visceral obesity), hyperlipidemia, and hypertension (20). In patients with T2DM, genetic signals mainly regulate β-cell development and function (18, 21) (**Figure 1**). Numerous GWAS confirmed that the variants markedly affect islet regulatory elements in the heritability of T2DM, and a large part of the association lies in the dysregulation of β-cell development and insulin secretion rather than disruption of insulin action on tissues (18, 22). In addition, a decline in the transcript encoding insulin in β-cells of patients with T2DM was also shown by single-cell RNA sequencing (23).

Environmental factors and hyperglycemia contribute to epigenetic changes in DNA and histones, modulating gene expression in organs implicated in the pathogenesis and progression of T2DM and β-cell function (24). Obesity leads to insulin resistance and may even result in early β-cell failure in some individuals who develop T2DM, and none of the drugs available is convincingly capable of preventing the gradual decline of β-cell function over time (18). Higher maternal BMI before pregnancy, greater calorie intake, more significant gestational weight gain, and maternal hyperglycemia are closely related to childhood obesity and T2DM. More importantly, maternal hyperglycemia and gestational DM are associated with precursors of T2DM (e.g., insulin resistance) in offspring, further indicating a powerful effect of maternal hyperglycemia on pancreatic β-cell development and function (25). Later in adulthood, the aging-associated decline in the β-cell responsiveness to carbohydrates partly explains the growing glucose intolerance with aging (26). As an effective therapeutic option for both obesity and DM, bariatric surgeries are beneficial far beyond contributing to weight loss but also recovering islet function by reversing metabolic disorders and normalizing the levels of glucagon-like peptide 1 (GLP-1) and peptide YY (PYY) (27).

### 3.2 Microenvironmental remodeling related to adiposity

Obesity is characterized by the overaccumulation of adipose tissue. The expansion of adipose tissue in non-adipose sites elevates the levels of certain metabolites, with the overproduction of inflammatory cytokines fueling systemic inflammation and disruption of cellular function, jointly contributing to impaired insulin signaling, damaged physiological and metabolic regulation, locally induced loss of β-cell function, the onset of hyperglycemia, and the eventual occurrence of DM.

#### 3.2.1 Ectopic expansion of adipose tissue

So far, three types of adipocytes have been identified in the human body (28). The subcutaneously stored and energy-producing white adipocytes are dominant and capable of secreting adipokines such as leptin, adiponectin, etc. In a much smaller amount, brown adipocytes located within supraclavicular, paravertebral, mediastinal, and other adipose-tissue depots can be activated and produce heat during cold exposure, with their activity being negatively correlated with age, BMI, and fasting glycemia, indicating the regulatory roles of brown adipocytes in cold-induced thermogenesis and energy metabolism (29). Scattered within white adipose tissue and characterized by their progenitor cellular origin, beige (thermogenic beige, or brown-and-white (“bright”)) adipocytes, as their names imply, can turn into brown adipocytes in response to cold exposure (30), exercise (31), and endocrine signals (32). Accordingly, different adipocytes induce different metabolic impacts on adiposity: the brown and beige adipocytes offer considerable benefits to energy homeostasis, whereas the altered white adipocytes in obesity are the culprit for multiple metabolic disorders (33). In detail, the changes in white adipocytes include alterations in size, function, inflammatory state, and whole-body distribution. On the cellular level, adipose tissue abnormalities are usually seen in the composition of extracellular matrix (ECM), vascularization, size, number of adipocytes, oxidative stress, adipokine secretion, and the inflammatory state of immune cell infiltration (34). As the adipose tissue expands exorbitantly with the increasing size of adipocytes, the cells become less responsive to insulin due to the reduced efficiency of glucose transport caused by stretched cell surface of enlarged adipocytes and oxidative stress caused by over-nutrition, a condition called insulin resistance that commonly exists in T2DM (35). Fundamentally, the excessive accumulation and expansion of white adipose tissue (WAT) is related to a remodeled microenvironment in obesity characterized by aberrant inflammation, fibrosis, hypoxia, dysregulated secretion of adipokines, and disrupted mitochondrial function (34, 36), which impairs insulin signaling, triggers insulin resistance, reduces insulin-stimulated glucose-transport activity (37), and speeds β-cell dysfunction, playing a pivotal role in the pathogenesis of T2DM. Besides, it was suggested that instead of the stage of obesity, the distribution of adipose tissue is more critical in developing insulin resistance (38).

**Figure 2:**
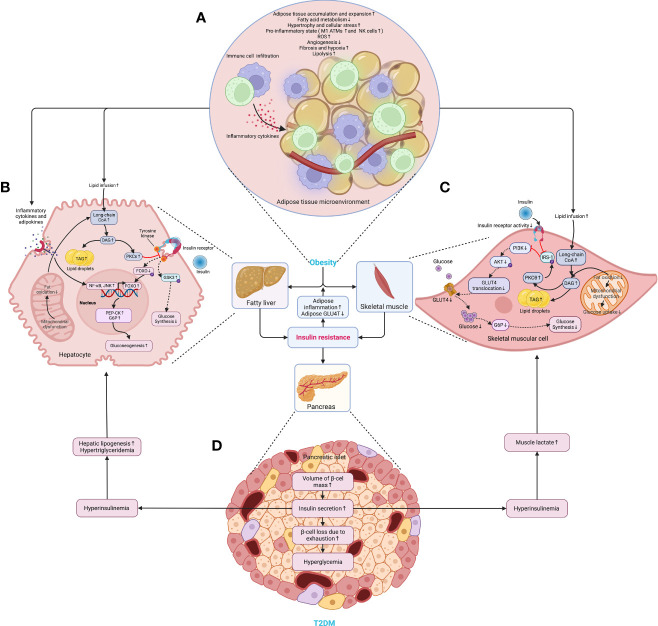
The critical roles of obesity-induced insulin resistance in the consequent pathogenesis of T2DM. **(A)** The aberrant accumulation and expansion of adipose tissue in obesity create a microenvironment characterized by hypoactive fatty acid metabolism and fuel cellular stress and pro-inflammatory perturbations, which results in an increased lipolysis, oxidative stress, and hypoxia due to fibrosis and insufficient angiogenesis. **(B)** In the liver, the excessive infusion resulting from enhanced lipolysis within adipose tissue causes a transient increase in DAG, which occurs once the rates of DAG synthesis exceed rates of mitochondrial long-chain CoA oxidation under mitochondrial dysfunction, and then the DAG is synthesized into TAG and stored as lipid droplets. Meanwhile, DAG activates PKCϵ and blunts the insulin receptor tyrosine kinase, damaging insulin signaling and decreasing insulin-stimulated glycogen synthesis owing to reduced phosphorylation of GSK3. At the same time, it also dampens the activity of glycogen synthase and indulges hepatic gluconeogenesis through decreased phosphorylation of FoxO, where the increased FoxO translocates to the nucleus and enhances the gene transcription of gluconeogenic enzymes, such as PEP-CK and G6P (39). Similarly, the cytokines and adipocytokines released in systemic inflammation increase hepatic gluconeogenic enzyme transcription by activating NF-κB and JNK. **(C)** In the skeletal muscle, the increased lipid fusion results in the accumulation of intracellular long-chain CoA and elevates the level of DAG, which is also caused by the decline in fat oxidation owing to mitochondrial dysfunction and subsequent reduction in glucose uptake. Apart from increasing the synthesis of TAG as cellular lipid storage, DAG also activates the theta isoform of protein kinase C (PKCθ), which leads to increased phosphorylation of IRS-1 and hinders insulin signaling and subsequent activation of the PI3K/AKT pathway, dampening glucose transport and glycogen synthesis. **(D)** While obesity-induced insulin resistance exacerbates inflammation and glucose transport in adipose tissue, the volume of β-cell mass is increased to meet the growing demand for insulin secretion. However, the increased insulin secretion results in hyperinsulinemia afterward and exasperates both hepatic and systemic lipid accumulation. Moreover, hyperinsulinemia increases the level of lactate in muscles, which is released into circulation and used as a substrate for hepatic lipogenesis. Finally, β-cells collapse, and the deficiency of insulin secretion leads to hyperglycemia. AKT, protein kinase B; ATM, adipose tissue macrophage; CoA, acetyl coenzyme A; DAG, diacylglycerol; FoxO, forkhead box subgroup O; G6P, glucose 6-phosphate; GLUT4, glucose transporter type 4; GSK3, glycogen synthase kinase 3; IRS-1, insulin receptor substrate 1; JNK, Jun N-terminal kinase; NF-κB, nuclear factor κB; NK, natural killer (cell); P, phosphorylation; PEP-CK, phosphoenolpyruvate carboxykinase; PI3K, phosphatidylinositol-3-kinase; PKCϵ (θ), epsilon (theta) isoform of protein kinase C; ROS, reactive oxygen species; TAG, triglyceride.

**Figure 3:**
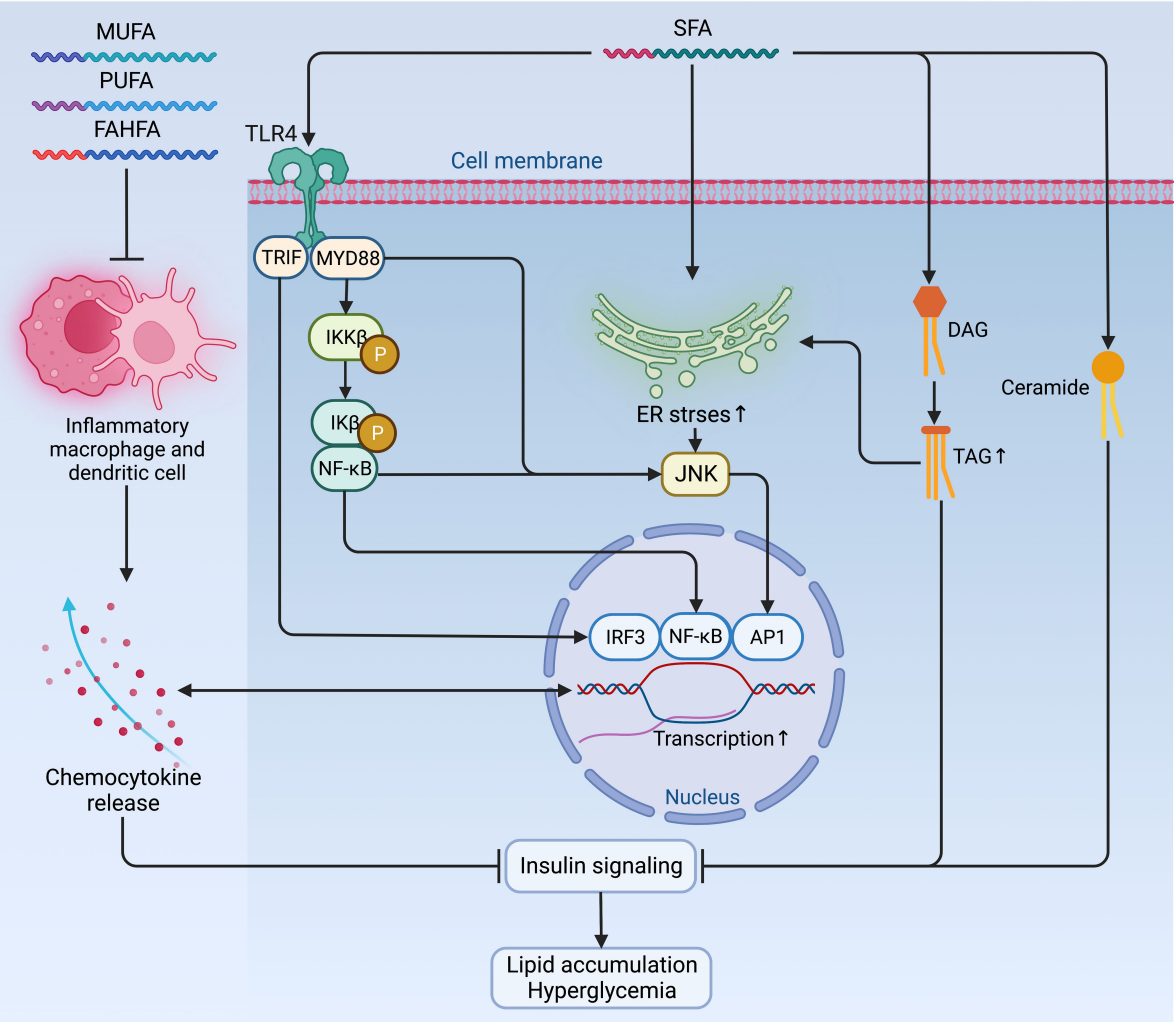
Mechanisms of fatty acids affecting insulin signaling and fueling hyperglycemia. Contradictory to the anti-inflammatory and insulin-sensitizing effect of PUFAs, MUFAs, and FAHFAs, SFAs sabotage insulin sensitivity by promoting pro-inflammatory signaling *via* TLR4 and its adaptor proteins TRIP and MYD88, which jointly enhance the activity of pro-inflammatory pathway and transcription factors, such as IRF3, NF-κB, and AP1 to augment the expression of chemocytokines. Mutually, these pro-inflammatory chemocytokines can also activate these pro-inflammatory transcription factors as a positive feedback loop to maintain an inflammatory environment detrimental to insulin signaling. Meanwhile, the accumulation of TAG, ceramides, and increased ER stress due to activated NF-κB signaling and inflammatory cascade can also exacerbate insulin resistance and fuel hyperglycemia. AP1, activator protein 1; ER, endoplasmic reticulum; FAHFA, branched fatty acid esters of hydroxy fatty acid; IKKβ, inhibitor of nuclear factor-κB (NF-κB) kinase subunit-β; Ikβ, inhibitor of NF-κB subunit-β; IRF3, interferon regulatory factor 3; JNK, Jun N-terminal kinase; MUFA, monounsaturated fatty acid; MYD88, myeloid differentiation primary response protein MYD88; NF-κB, nuclear factor-κB; P, phosphorylation; PUFA, polyunsaturated fatty acid; SFA, saturated fatty acid; TAG, triglyceride; TLR4, Toll-like receptor 4; TRIF, TIR-domain-containing adaptor-inducing interferon-β.

Later, the volume of the liver, skeletal muscles, other organs, and tissues increases following the accumulation of WAT, and so does the β-cell mass (28). In particular, the WAT expansion in skeletal muscles and the liver increases the level of diacylglycerol (DAG), which activates the epsilon form of protein kinase C (PKCϵ), leading to decreased glucose uptake in muscles and disrupts insulin-induced activation of glycogen synthesis and suppression of glucose production in the liver, fueling hyperglycemia (39). Moreover, the expansion of adipose tissue in the liver leads to steatosis, exacerbating insulin resistance by causing lipotoxicity-induced cellular dysfunction and apoptosis (40). Once present, which is very common in obesity, β-cells secret more insulin to maintain normal glycemic homeostasis. However, the concentration of blood glucose increases following the piecemeal failure or diminish of β-cells (**Figure 2**). Of note, the obesity-induced transition from insulin resistance to T2DM involves dysfunction of both pancreatic α- and β-cells resulting in upregulation of hepatic gluconeogenic gene transcription with the cytokines and adipocytokines released in systemic inflammation suppressing insulin action (will be discussed later), which in turn, increases hepatic gluconeogenic enzymes transcription through the activation of nuclear factor κB (NF-κB), Jun N-terminal kinase (JNK), and ceramide biosynthetic pathways (39) (**Figure 3**).

Meanwhile, hypoxia, fibrosis, and mitochondrial dysfunction in the microenvironment of obesity also contribute to the development of DM (**Figure 2**). Hypoxia, an early determinant and initiator of ECM disruption and fibrotic stress, is a consequence of insufficient angiogenesis owing to fast and excessive adipose tissue expansion, over-activating the transcription factor hypoxia-inducible factor 1α (HIF-1α) that triggers fibrosis, enhances local inflammation and lowers insulin sensitivity (34, 41). Fibrosis, on the other hand, refers to a pathological process resulting from an abnormality in the composition of ECM, which restrains the lipid storage of adipocytes and promotes lipotoxicity (34), a detrimental effect of excess fat accumulation on glucose metabolism and metabolic pathways, whether in adipose tissue or peripheral organs, such as liver, heart, pancreas, and muscles, which impairs insulin signaling and pancreatic β-cell function (42). Many types of lipids, namely non-essential fatty acids (NEFAs), triacylglycerol (TAG), DAG, and ceramides, etc. (will be introduced below), and their components as well as their localization within cells, jointly contribute to lipotoxicity (42). Consequently, lipotoxicity induces mitochondrial dysfunction, mainly involving a reduction in mitochondrial mass and ATP production with overproduction of reactive oxygen species (ROS), which is detrimental to insulin sensitivity due to the critical role of mitochondria in regulating the adipogenesis, fatty acid synthesis, esterification, and lipolysis of adipocytes (36, 43). In this context, certain metabolites, such as the elevated levels of free fatty acids (FFAs) resulting from enhanced lipolysis, for instance, may increase endoplasmic reticulum (ER) stress and impair β-cell function (18). Nevertheless, the expansion of adipose tissue cannot be thoroughly repudiated despite being intensively associated with various adverse outcomes since ample evidence has shown that adipose tissue expanding in a “healthier” way can be protective and improves the prognosis and survival of patients in many medical conditions like cardiovascular disease and cancer, a so-called “obesity paradox” (44, 45).

#### 3.2.2 Nutrients and metabolites

The contribution of obesity to insulin resistance is beyond the impairment of insulin signaling but involves the interplays of various metabolic pathways and essential nutrients and metabolites. Some nutrients and metabolites can directly impact insulin signaling by regulating the components of the insulin signaling pathway or indirectly mediate the substrates flux of metabolic pathways such as lipogenesis, lipid oxidation, protein synthesis, degradation, hepatic gluconeogenesis, and the post-translational modulation of proteins (46).

##### 3.2.2.1 Fatty acids (FAs)

Whether endogenously synthesized or ingested from the diet, the excessive accumulation of lipids is very common in obesity. However, the jeopardizing effects of lipids on insulin sensitivity differ depending on their biochemical classification. Among fatty acids (FAs), saturated fatty acids (SFAs) induce negative impacts on insulin sensitivity by promoting pro-inflammatory signaling via Toll-like receptor 4 (TLR4) (47) and enhancing the synthesis of DAG and ceramides (48). Moreover, SFAs also increase ER stress by activating NF-κB signaling and inflammatory cascade to exacerbate insulin resistance in metabolic organs and immune cells (46, 49, 50). In contrast, the polyunsaturated fatty acids (PUFAs), monounsaturated fatty acids (MUFAs), and branched fatty acid esters of hydroxy fatty acids (FAHFAs) are shown to have an anti-inflammatory and insulin-sensitizing effect in animals, although this effect is marginal in humans in a dietary way (46, 51–54) (**Figure 3**).

Other types of FAs are also implicated in the regulation of insulin sensitivity. Since the short-chain fatty acids (SCFAs) are released by the gut microbiome, the alteration of microbiome composition in obesity and T2DM disrupts SCFA production and affects insulin sensitivity and energy metabolism. Usually, SCFAs effectively suppress appetite by stimulating the secretion of gastrointestinal (GI) peptides (e.g., leptin, PYY, and GLP-1) (55–57) and activating G protein-coupled receptors (GPCRs) in the brain (58). Moreover, SCFAs also activate the insulin-sensitizing intestinal gluconeogenesis that promotes satiety and increases energy expenditure via the periportal neural system (59). Finally, with their roles in suppressing lipolysis and increasing oxidative metabolism in WAT, liver, and skeletal muscles (60), SCFAs ameliorate inflammation in adipose tissue (55) and improve insulin signaling in rodents. However, the beneficial effect of SCFAs on humans is still unclear.

Other lipids, such as DAG, an immediate precursor of TAG elevated in muscles and liver of patients with obesity and T2DM, have been associated with declined insulin sensitivity through dysfunctional modification of insulin signaling molecules (61). Nevertheless, the higher levels of DAG and worse insulin sensitivity are not definitely correlated (62). Likewise, elevated levels of ceramides of a certain chain length and saturation of fatty acids, which belong to sphingolipids, are reported to impair insulin sensitivity through reduced adiponectin and increased inflammation caused by insulin resistance initially (63, 64). Technically, the specific structures instead of the ceramide levels affect insulin sensitivity (65). For example, overexpression of ceramide synthase 2 (CERS2), a catalyst for the synthesis of very-long-chain fatty acids (VLCFAs) in hepatocytes, enhances insulin signaling despite elevating the total level of ceramide (66), whereas the depletion of CERS2 in WAT and liver results in weight gain and deterioration of insulin resistance (67). In addition, as the major components of the cellular membrane, certain phospholipid species also regulate insulin sensitivity by influencing the mitochondrial function, inflammation, production of BAs, and FAs uptake (46).

Furthermore, insulin resistance in obesity is also a direct outcome of disrupted metabolic substrate shift for oxidation between glucose and FAs (68), with the over-accumulation of lipids resulting from dietary intake or lipogenesis being the main driver. At the same time, the increased lipolysis further boosts the release of FAs and glycerol, contributing to systemic inflammation in a mutual way (69). As a result, the increased lipolysis caused by insulin resistance elevates the deposition of lipids and their flux in the skeletal muscles and liver, leading to mitochondrial dysfunction in muscles and enhanced gluconeogenesis in the liver, favoring an exacerbated insulin sensitivity and rise in glycemic levels (**Figure 2**).

##### 3.2.2.2 Amino acids (AAs)

Like many other metabolites, the levels of amino acids (AAs) are altered in patients with obesity and insulin resistance with their capability of increasing the risk or deteriorating T2DM (70, 71). Yet positively, they serve as the markers in differentiating the stage of DM (72). In particular, the branched-chain amino acids (BCAAs) are the most studied ones and are suggested to be associated with obesity-related metabolic syndrome and cardiovascular risk (73). Initiated by insulin resistance in the brain (74) and increased WAT inflammation, ER stress, hypoxia, and mitochondrial dysfunction, the increase of BCAAs is caused by the declined expression of BCAA catabolic enzymes and suppressed oxidation in WAT of both animals and humans with obesity, insulin resistance and T2DM (46, 71), where the dampened oxidation in adipose tissue and liver leads to excessive lipid release and shunted oxidation of BCAAs in skeletal muscles, resulting in accumulation of lipid and jointly boost lipotoxicity that contributes to impaired insulin signaling and development of T2DM (71). Meanwhile, the increase in the BCAA-producing bacteria in the gut microbiome and the decreased hepatic BCAA catabolism contribute to the increased BCAA levels (75, 76). However, whether the elevated BCAA levels lead to T2DM is currently elusive. In addition, high levels of methionine and circulating aromatic amino acids (AAAs) are also involved in insulin resistance and T2DM. While the methionine restriction is related to weight loss, improved energy intake, insulin sensitivity, and adiponectin secretion, the elevation in the levels of AAAs is shown to be related to the over-ingestion from diet and over-production by the *Escherichia coli* owing to the excessive supply of glucose (77).

##### 3.2.2.3 Other metabolites

Some metabolites affect insulin sensitivity through post-translational modification, such as acetyl-CoA and palmitate, mediating lipid and glucose metabolism through acetylation and palmitoylation (46). And nucleotides, especially the levels of uridine, are related to insulin resistance and increased in patients with T2DM (78), which requires further studies to elucidate the role of nucleotides in the pathogenesis of obesity and T2DM.

#### 3.2.3 Systemic inflammation

Triggered by excessive lipid species, the elevation of circulating lipopolysaccharide (LPS), hypoxia, and fibrosis, the consequent occurrence of systemic inflammation in obesity plays a causative role in insulin resistance, insulin deficiency, and dysregulation of energy homeostasis. The obesity-induced low-grade inflammation affects many organs and involves activation of the innate immune system that impacts metabolic balance, resulting in tissue damage *via* increased fibrosis and necrosis at the same time (79). While locally, the gradual deterioration of inflammatory reaction within pancreatic islet leads to the loss of β-cell mass and cellular dysfunction and the eventual onset of T2DM (8).

In the shade of the dysregulated secretion of pro-inflammatory cytokines, including tumor necrosis factor (TNF), interleukin (IL) −1β, IL−6, IL−8, leptin, resistin, and monocyte chemoattractant protein 1 (MCP1) by adipocytes and macrophages within adipose tissue, the low-grade systemic inflammation dampens insulin signaling in the conjunction of decreased anti-inflammatory cytokines and adipokines such as IL−10 and adiponectin as the result of tissue remodeling fueled by adipocyte apoptosis (34, 80, 81). In this process of activating the innate immune system, the overall number of macrophages and its ratio of M1 to M2 phenotype increases as a response, which also involves many other immune cells such as T cells, B cells, and natural killer (NK) cells, etc. (82).

In addition, the increased infiltration of macrophages in the liver damages hepatic insulin sensitivity in collaboration with inflammatory chemokines and cytokines (83). Apart from worsening the insulin resistance of the distant sites (e.g., liver and skeletal muscle) (84), inflammation is another major factor contributing to the transition from insulin resistance to weakened glucose tolerance by inducing lipolysis that increases the delivery of fatty acid flux and glycerol to the liver, enhancing gluconeogenesis and compromising the function of α- and β-cells by worsening glucolipotoxicity, hyperglycemia and inflammation itself (39, 84–87) (**Figure 2**). Mechanistically, as the predominant regulator, the enhanced activation of NF−κB, as well as other specific protein kinases (inhibitor of NF-κB kinase subunit-β [IKK-β], IKK-ϵ, Jun N−terminal kinase [JNK] and protein kinase Cγ [PKCγ]), interfere with insulin signaling in both direct and indirect manners (88, 89).

Overall, the early-phase inflammation in obesity is triggered as a positive and adaptive response to anabolic pressure fueled by energy imbalance, or broadly, an intricate immunometabolic crosstalk tailored to sustain tissue integrity and homeostasis (8). Unfortunately, the long-term chronic inflammation and excessive adipose tissue expansion eventually contribute to the development and progression of T2DM by promoting insulin resistance, fibrosis, adipocyte dysfunction, and cell death (34).

### 3.3 Autophagy

**Figure 4:**
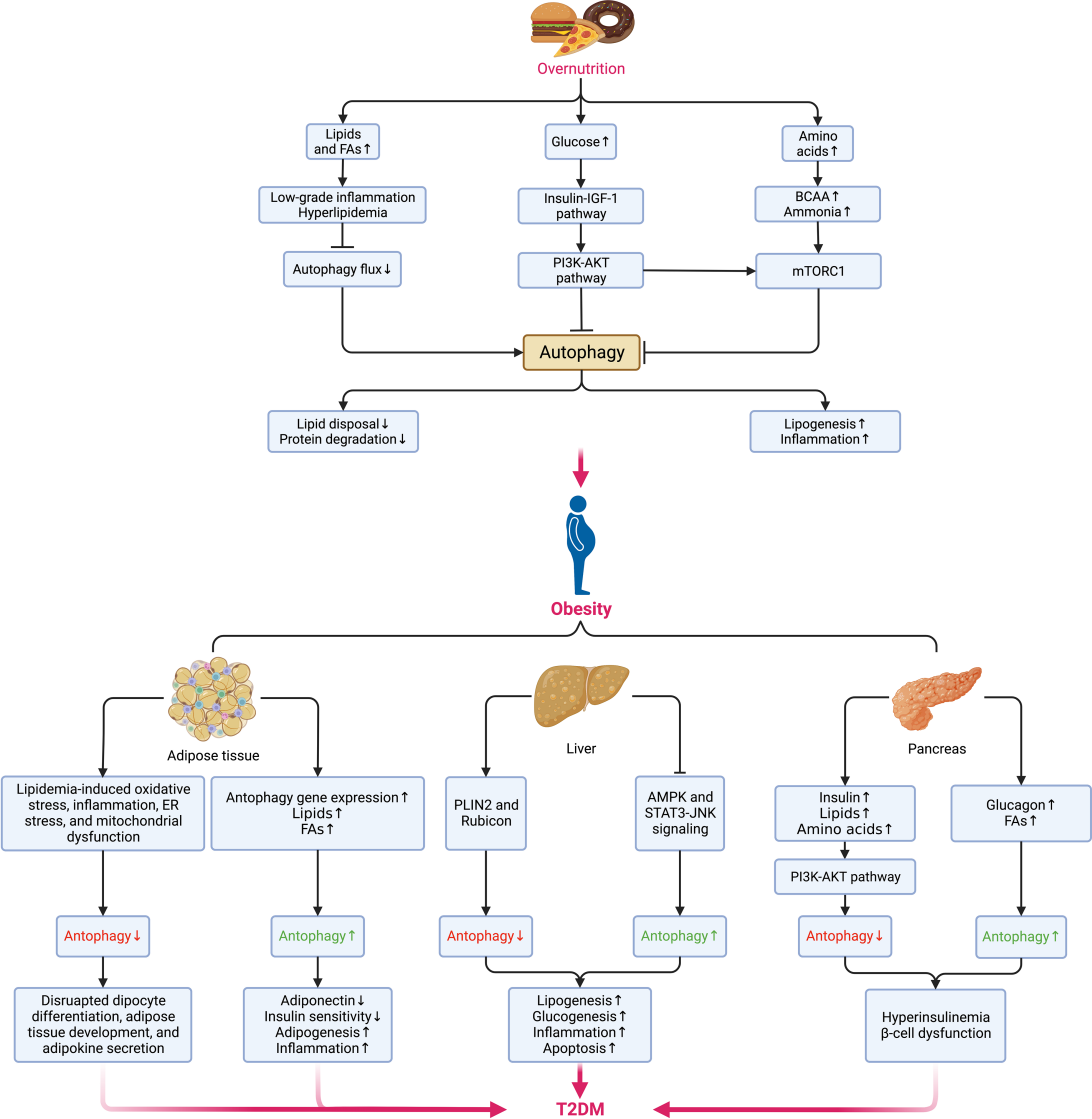
Alterations of autophagy in different metabolic organs during the transition from overnutrition and obesity to T2DM. The excessive intake of nutrients such as lipids, glucose, and amino acids results in the suppression of autophagy via different signaling pathways and contributes to obesogenesis by increasing the accumulation of lipids, and proteins, enhancing low-grade systemic inflammation and exacerbating insulin signaling. In obesity, distinct dissimilarities can be observed in the changes in autophagy among different sites. In adipose tissue, the elevations in the levels of lipids and FAs and the upregulation of autophagy genes can enhance autophagy, whereas cellular stress can suppress autophagy. While the hepatic can also be enhanced or blunted by different signaling pathways and lead to the promotion of lipogenesis, glucogenesis, inflammation, and apoptosis. Insulin and metabolites, such as lipids, amino acids, glucagon, and FAs can also induce a dual impact on pancreatic autophagy and result in hyperinsulinemia as an initial protective mechanism against hyperglycemia but eventually favors the onset of insulin resistance and DM following the concurrent dysfunction of β-cells. Pancreatic autophagy might also be enhanced by elevated levels of free fatty acids (FFAs) and glucagon in obesity. Jointly, all these disruptions in autophagy of different sites contribute to the aberrant accumulation of protein aggregates, lipids, and other detrimental components in the microenvironment that fuels cellular stress and causes insulin resistance and subsequent transition from obesity to DM. BCAAs, branched-chain amino acids; NAFLD, nonalcoholic fatty liver disease; NASH, nonalcoholic steatohepatitis. AKT, protein kinase B; AMPK, AMP-activated protein kinase; BCAA, branched-chain amino acid; DM, diabetes mellitus; ER, endoplasmic reticulum; FAs, fatty acids; IGF-1, insulin-like growth factor 1; JNK, Jun N-terminal kinase; mTORC1, mechanistic target of rapamycin (mTOR) complex 1; PI3K, phosphatidylinositol-3-kinase; PLIN2, perilipin 2; STAT3, signal transducer and activator of transcription 3.

Autophagy maintains cellular quality and organ function as a key but conserved homeostatic process via the disposal and recycling of cellular components while eliminating hazardous cells containing potentially toxic proteins, lipids, and organelles, which is mainly regulated by the mechanistic target of rapamycin (mTOR) kinase and the autophagy-related protein (ATG) family, with its aberrance (whether enhanced or suppressed) being involved in multiple metabolic disorders and diseases, including obesity, T2DM, and cancer (90, 91). Due to its crucial physiological function, autophagy is highly sensitive to changes in nutrients, energy status, microenvironment (hypoxia, oxidative stress, DNA damage, and protein aggregates), cellular metabolism, and intracellular pathogens (92). Generally, the alteration of autophagy (either site-dependently enhanced or suppressed) in the context of obesity is complicated since they depend on various conditions (93) (**Figure 4**), where the hyperactivation of mTOR under overnutrition leads to the suppression of autophagy owing to the imbalance between calorie intake and energy expenditure (94). However, most existing experimental data suggest that autophagy is enhanced in the adipose tissue (90).

Oppositely, suppressed autophagy might disrupt adipocyte differentiation, adipose tissue development, and adipokine secretion regarding its critical roles in regulating adipose tissue development, adipogenesis, and lipid metabolism (90, 95). Besides, the expansion of adipose tissue increases the secretion of pro-inflammatory adipokines while decreasing the adiponectin level. As a result, autophagy is upregulated, accompanied by the elevated expression of autophagy genes in adipose tissue as a compensatory anti-inflammatory response (96). Yet, the possibly increased intracellular lipid disposal and decreased lipolysis and proteolysis under enhanced autophagy can cause visceral fat accumulation and insulin resistance (93, 97). Even so, it would be hasty to say either the alteration of autophagy is a cause of obesity or vice versa based on the evidence so far. If it is, hypothetically, we can assume that the dysfunction of autophagy contributes to excessive adipose tissue expansion, low-grade inflammation, and metabolic dyslipidemia, thereby resulting in hyperinsulinemia and the onset of T2DM. Meanwhile, the residence of non-adipocyte cells in adipose tissue might also perturb autophagy in obesity under a conversed condition. For example, despite autophagy being enhanced in adipose tissue to rid lipid droplets and promote lipid catabolism in non-adipose tissues, autophagy in macrophages is somehow suppressed at the price of adipose tissue dysfunction (98).

Several common abnormalities in the microenvironment of obesity impairing insulin sensitivity, such as lipid accumulation, oxidative stress, inflammation, ER stress, and mitochondrial dysfunction, induce a negative impact on autophagy as well (99). Given the suppression of autophagy in overnutrition, the downregulated secretion of glucagon under nutrient excess leads to hyperinsulinemia that blunts autophagy, compromising insulin signaling in return and boosting the development of T2DM (100). During this process, the protective autophagy in pancreatic β-cells is essential in maintaining metabolic homeostasis (101). Unfortunately, the excessive autophagy regulating β-cell death may deteriorate β-cell loss under cellular stress and fuse the onset of T2DM (102). As insulin resistance can be directly affected by autophagy in insulin-sensitive tissues such as adipose tissue, skeletal muscles, pancreas, liver, and the brain (103), the ectopic expansion of adipose tissue in these sites disrupts autophagy and accumulates dysfunctional organelles, promoting tissue-specific insulin resistance and damaging pancreatic function. Meanwhile, pathophysiological changes occurring during insulin resistance might reciprocally disrupt autophagy reciprocally (90). Finally, following the exacerbation of autophagy dysfunction, accumulation of ROS, and mitochondrial damage, the gradually aggravated insulin resistance leads to the occurrence and development of T2DM.

Beyond the influence on insulin sensitivity of different tissues and organs through complex mechanisms, autophagy can also be affected by inflammation in obesity. A variety of highly active adipokines and activated signaling pathways (e.g., JNK–activator protein 1 [AP1] complex and NF-κB) in different tissue types of low-grade inflammation can affect different autophagy responses (79). Through the activation of calpain or mTOR and inhibition of AMP-activated protein kinase (AMPK) in tissues, including heart and adipose tissue, obesity enhances pro-inflammatory response by decreasing autophagy flux (104). Interestingly, many cytokines or adipokines released during low-grade inflammation also enhance autophagy. In turn, autophagy inhibits the inflammatory response by scavenging damaged organelles (such as mitochondria) or intracellular pathogens and clearing pro-inflammatory complexes (104, 105), so it was proposed that inflammasomes are the central pivot connecting inflammation and autophagy coordination in obesity (90).

Overall, obesity causes a series of metabolic abnormalities, such as T2DM, cardiac dysfunction, hypertension, nonalcoholic fatty liver disease (NFLD), and polycystic ovary syndrome (PCOS) by fueling autophagy disruption (90). And inspired by the beneficial effect of autophagy modulation in the prevention and treatment of obesity and related metabolic disorders via lifestyle modification (such as exercise and calorie restriction) and pharmacotherapy, numerous preclinical studies have been carried out over the years. Nevertheless, the relationship between obesity, obesity-related metabolic abnormalities, and autophagy is complex. Currently, there are many elusive and even contradictory findings, as the changes in autophagy can be highly heterogeneous depending on the organ, metabolic status, space, and local inter-organ communication. Since both the activation and inhibition of autophagy are beneficial in improving metabolic disorders, it isn’t easy to fully justify the roles of autophagy in obesity-related T2DM onset, which warrants more future research in this field.

### 3.4 β-cell failure in pancreatic islet

**Figure 5:**
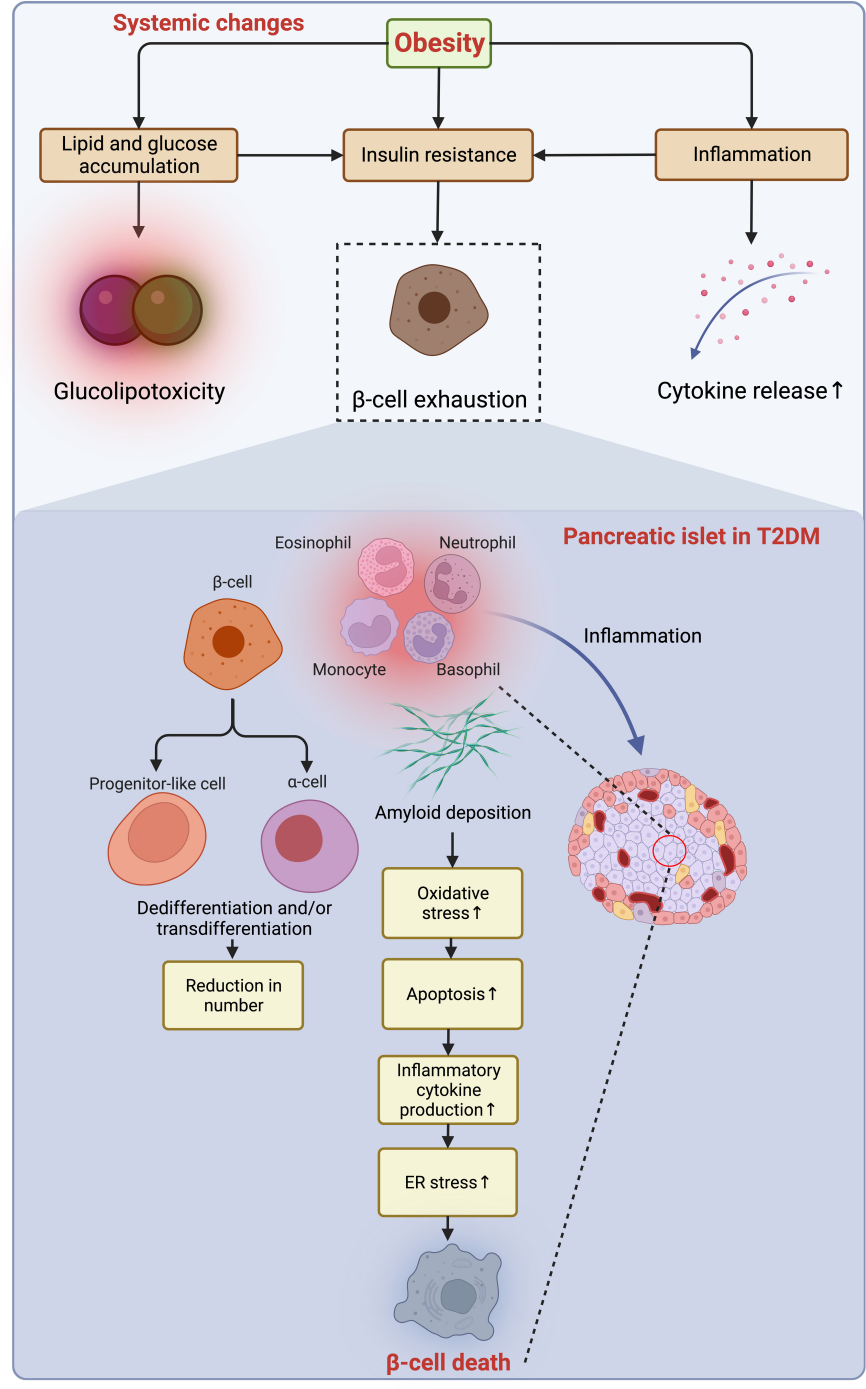
Obesity-induced acceleration of β-cell loss in the pancreatic islets of T2DM. The accumulation of lipid and glucose induces glucolipotoxicity, which jointly exacerbates insulin resistance and exhausts β-cells with enhanced low-grade inflammation owing to the increased secretion of pro-inflammatory cytokines to the microenvironment. In the islets of T2DM with a similarity to the inflammatory microenvironment as in adipose tissue, the deposition of amyloid worsens oxidative stress, resulting in increased apoptosis of β-cells, while the dedifferentiation of β-cells to progenitor-like cells or transdifferentiation to other identities (e.g., α-cells) further jeopardize the β-cell population. Collaboratively, the constant pro-apoptotic and pro-inflammatory signals promoting ER stress lead to β-cell loss. ER, endoplasmic reticulum; T2DM, type 2 diabetes mellitus.

Following the slow progression of T2DM, β-cells suffer from unbearable stress and deteriorated apoptosis (18) (**Figure 5**). Meanwhile, the number of macrophages is mildly increased in the islet of T2DM, and the β-cell loss (~40% reduction) is caused by multiple factors, including glucolipotoxicity and amyloid deposition enhancing β-cell apoptosis through oxidative and ER stress (18, 106, 107). The transition from obesity and insulin resistance to T2DM is triggered by β-cell failure, impaired glucose-stimulated insulin secretion (GSIS), and loss of β-cell function that is independent of cell loss in T2DM (18, 108), which is fueled by gradual dedifferentiation of β-cells to endocrine progenitor-like cells or transdifferentiation to other cell types (109, 110). As introduced, T2DM progresses in a much slower manner because of a much longer duration of loss of residual β-cell function and mass, even though the β-cell aging and senescence are accelerated by obesity (18). More importantly, this loss cannot be endogenously compensated with newly generated β-cells owing to the incapability of neogenesis and replication of the human pancreas after age thirty (111). However, now a variety of therapeutic approaches have been developed to reverse this process and are being tested and validated to treat both T1DM and T2DM by reactivating the neogenesis and regeneration of β-cells (112, 113), and some of these emerging measures are promising and may favor a cure for those who suffer from DM in the future.

Driven by long-term exposure of the islets to excessive nutrients and a constitutive increase in hormone synthesis and secretion, ER stress is another factor that adversely affects β-cell function and survival in obesity (108). To compensate for the insulin resistance in peripheral tissues, the demand for insulin synthesis is increased in obesity, where the excessive nutrients intake overloads ER protein folding capacity and thereby activates unfolded protein response (UPR) and PKR-like ER-associated kinase (PERK), resulting in inhibition of protein translation and insulin deficiency eventually (108). Likewise, prolonged exposure to hyperglycemia and hyperlipidemia of islets induces glucolipotoxicity that blunts insulin secretion and activates β-cell apoptosis (107). Meanwhile, ER stress leads to β-cell dysfunction by hindering normal synthesis and secretion of insulin and triggers ER-associated degradation of proteins and autophagy. A prolonged ER stress induces dedifferentiation and apoptosis of β-cells (114, 115). In this context, the short-term effective ER stress response made to maintain organelle homeostasis is of great importance for the development, function, and survival of β-cells (114). Of note, hyperactivation of the ER stress response can even be harmful to β-cells in the context of lipid overload (18). On the contrary, as a preferable strategy targeting obesity and T2DM, low-calorie Mediterranean-style or low-carbohydrate dietary regimens ameliorate insulin resistance, insulin clearance, and β-cell function (116).

### 3.5 The microbiome-gut-brain axis

**Figure 6:**
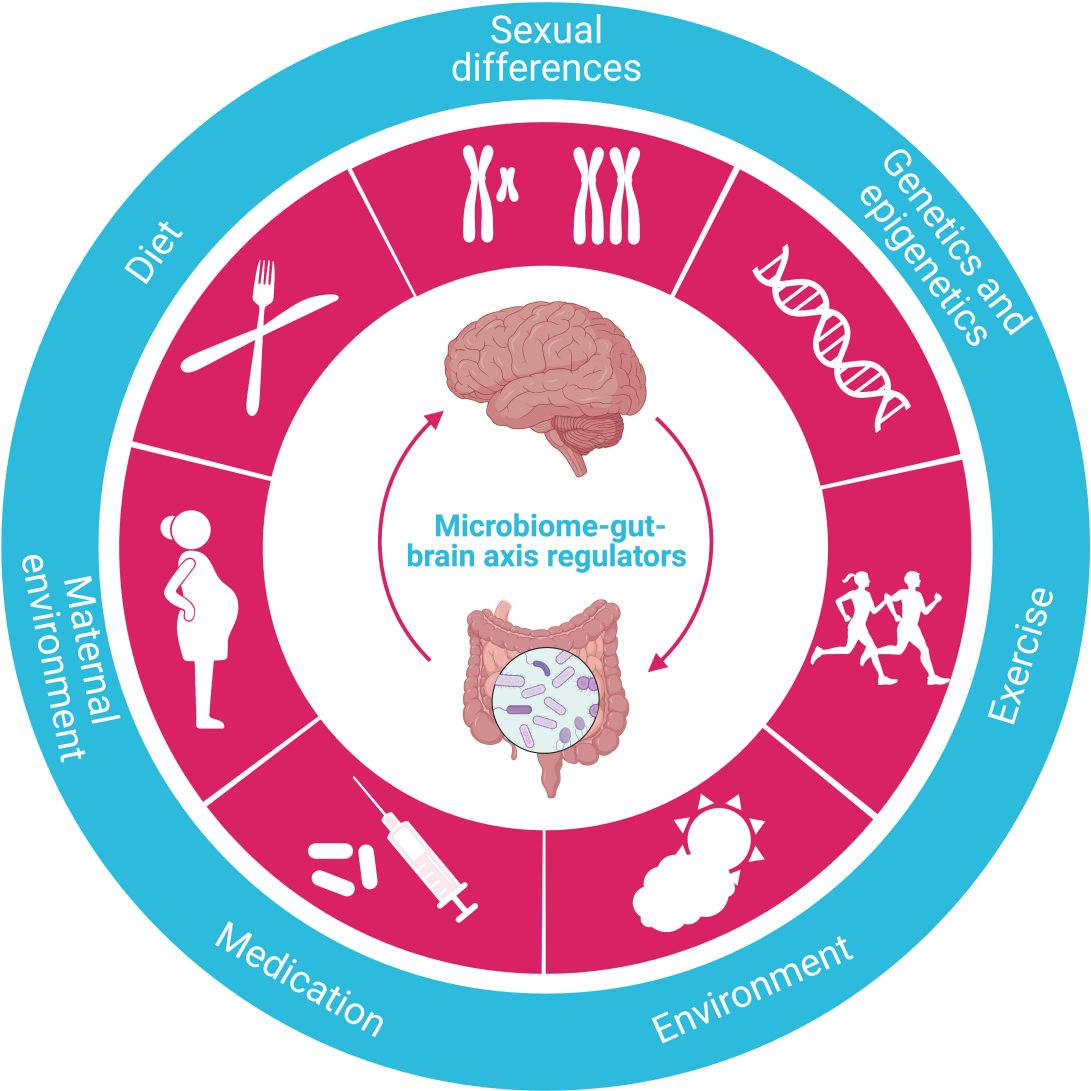
Regulators of the microbiome-gut-brain axis. The crucial function of the microbiome-gut-brain axis is regulated by various factors, such as diet, sexual differences, genetics/epigenetics, exercise, environment, medication, and maternal environment.

Regulated by various factors, the microbiome-gut-brain axis is one of the most critical mechanisms regulating whole-body metabolism, adiposity, energy balance, and central appetite and food reward signaling in humans (**Figure 6**). The aberrance of this axis is closely associated with several metabolic diseases, including obesity and T2DM (117). Significantly, microbiome dysfunction is the main culprit responsible for energy imbalance, fat deposition, inflammation, insulin resistance, glucolipotoxicity, and dysregulation of endocrine signaling pathways by either direct or indirect effect (118).

Harboring about 100 times more genetic information than humans (119), the gut microbiome is dominated by five bacterial phyla: *Actinobacteria*, *Bacteroidetes*, *Firmicutes*, *Proteobacteria*, and *Verrucomicrobia*. In general, the high diversity of the microbiome is crucial in maintaining a healthy physical condition. The composition of the microbiome is both endogenously and exogenously shaped by multiple factors, including maternal environment, age, ethnicity, diet, medication (especially antibiotics), and genetics (120), among which the diet is predominately determinative, and the composition of gut microbiome changes with the development of obesity and T2DM. Similar to studies in rodents, an increased ratio of *Firmicutes*/*Bacteroidetes* was observed in humans with obesity and suggested to be associated with more dietary energy extraction (121). In addition, the decrease in the diversity of the microbiome is related to the increase in BMI, fat mass, inflammatory markers, reduced insulin sensitivity, and dyslipidemia (122), and changes in the composition of the microbiome also affect fasting glucose, glycated hemoglobin (HbA1c) levels, and T2DM development by inducing significant alterations such as changes in GI peptides, appetite, inflammation, insulin resistance, fat storage, hepatic lipid metabolism, and hyperglycemia by converting dietary nutrients into metabolites thus regulating their central and peripheral effect (118, 123–125). Overall, the gut microbiome affects metabolic processes and insulin signaling by regulating inflammation and the production and disposal of metabolically effective components (126, 127).

Although evidence from rodent studies has provided plenty of valuable connections between the intestinal microbiome and host health as well as the development of metabolic diseases, these results must be interpreted with caution due to the essential differences between mice and humans exist in GI anatomy, genetic background, metabolic phenotype, along with more confounding factors in human such as diet, lifestyle, medication history, etc. The changes in gut microbial production (e.g., metabolites) or host exposure to bacteria-derived components (e.g., endotoxin) have been suggested to induce a more considerable impact on the development of metabolic disorders compared to its direct influence on the metabolic process resulting from its altered composition (128). Furthermore, rodent models indicated that the gut microbiome is vital in increasing inflammatory tone in obesity and T2DM (129). The LPS-induced endotoxemia, as demonstrated in mice and consistent with humans, is responsible for the inflammatory status (130, 131). While under normal conditions, the gut barrier only allows a minimal amount of LPS to enter the circulation, the elevated LPS in obesity and DM binds to TLR4 and activates NF-κB signaling pathways, promoting the secretion of inflammatory cytokines like IL-1, IL-6, and TNF-α (132, 133). Hence, it can be hypothesized that the endotoxins produced from the GI tract may flux into the liver through the portal vein, which would probably deteriorate the inflammation and insulin signaling in the liver. What is worse, the diet-induced increase in gut permeability and subsequent reduction in protective gut barrier function contributes to the development of T2DM owing to the increased circulating levels of bacterial DNA (134, 135). Functionally, the gut microbiome produces numerous organic compounds such as nitric oxide, ammonia, carbon oxide, indole, and hydrogen sulfide that could be pro- or anti-inflammatory and influence gut permeability (136), thereby modulating systemic or local metabolism.

As the primary workforce of metabolite generation in the GI tract, the microbiome mediates metabolic processes such as enteroendocrine regulation, GLP-1 secretion, inflammatory response, glucose uptake, FA oxidation, and energy metabolism via the production of SCFAs and SCFA-mediated activation of GPCRs. Interestingly, the levels of SCFAs are increased in patients with obesity, which seems contradictory to its benefits on metabolic control. The truth is, the increased relative abundance of SCFA-producing microbiome and increased SCFA content in obesity is veiled by the rapid absorption by the host and the gut microbiome itself, resulting in the incompetence of SCFA disposal (120). In contrast, obese patients with T2DM have been shown to have a relatively reduced number of SCFA-producing bacterial species (137).

Bile acids (BAs), on the other hand, participate in the maintenance of human health and the development of metabolic diseases as signaling molecules activating receptors in the gut, liver, and adipose tissue (138), majorly under the modulation of the nuclear Farnesoid X receptor (FXR) and its downstream targets fibroblast growth factor (FGF) 15/19 in intestines and small heterodimer partner in liver (139). Beyond its synthesis and modifications of BAs after secretion (140), the gut microbiome also regulates the uptake of BAs (141). Furthermore, BAs have been shown to promote GLP-1 secretion (142) and control the expression and activity of genes involved in BA, lipid and carbohydrate metabolism, energy expenditure, and inflammation (46, 143, 144). A bidirectional effect exists between the gut microbiome and BAs, and dysregulation of BA metabolism in conjunction with dysbiosis contributes to the metabolic disorder in obesity and T2DM (143). The BA metabolism, total concentration, and pool composition are altered in obesity and T2DM, leading to aberrance in energy balance, lipid metabolism, glucose metabolism, and immune function (143). Given the ample evidence of bariatric surgeries recovering metabolic homeostasis via the normalization of BA metabolism, BAs are now deemed amendable targets to treat metabolic disorders with different therapeutic strategies.

Concerning the roles of the neurological system in metabolic imbalance. Fundamentally, the level of glycemia is strictly regulated by the nervous system: 1) the central nervous system (CNS), particularly the hypothalamus, detects the glycemic alterations throughout the day in blood, and 2) the autonomic nervous system (ANS), including the enteric nervous system (ENS) and the vagus nerve (VN), detect the glycemic variations within the whole GI tract, from the mouth to the colon, and the portal vein during post-prandial period (126). And the secretion of glucose-lowering and elevating hormones and peptides will be tuned according to the level of blood glucose. This joint and balanced mediation is crucial in maintaining systemic metabolic homeostasis. Its dysregulation results in extensive alterations, notably in insulin signaling and glucagon action controlled by metabolic, endocrine, and paracrine regulatory mechanisms. Insulin enhances anabolic processes by promoting the storage of metabolic fuels, while the catabolic effects of glucagon oppose it. The intestines secret various peptides that act on the pancreatic islets, specifically the GLP-1 and glucose-dependent insulinotropic polypeptide (GIP), commonly known as incretins, to stimulate insulin secretion while suppressing glucagon production (145).

**Figure 7:**
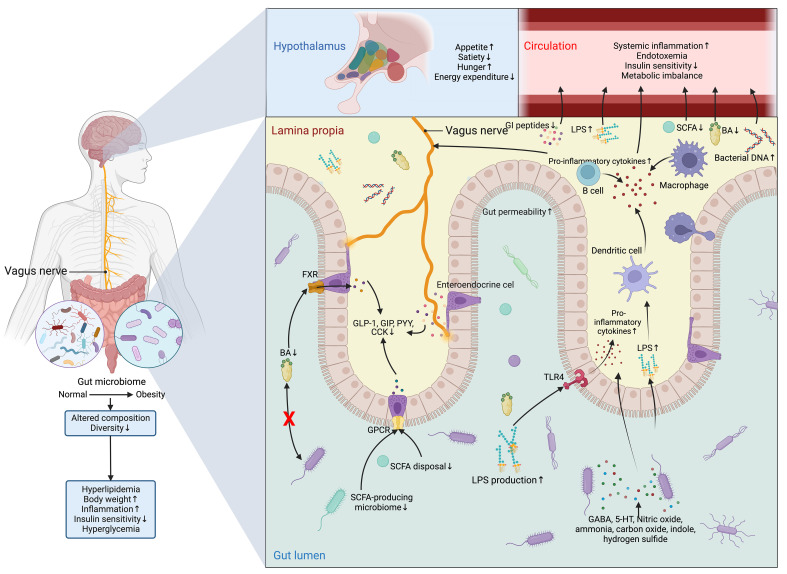
Dysfunction of the microbiome-gut-brain axis in obesity and T2DM. The composition and diversity of the gut microbiome are significantly altered in obesity. Apart from the consequent aberrances in the metabolism and metabolites of the microbiomes, such as the decrease in the production of BAs and SCFAs and the increase in LPS, the protective function of the gut is compromised by the increased permeability resulting from the inflammatory stimulations of nitric oxide, ammonia, carbon oxide, indole, and hydrogen sulfide, allowing the drastic elevation in the flux of LPS, which can not only activate the TLR4 on enterocytes to promote the secretion of inflammatory cytokine and recruit inflammatory dendritic cells, B cells, and macrophages, but also directly inducing the production of inflammatory cytokines from these cells. Meanwhile, the profound reduction in the production of SCFAs and BAs leads to the subsequent drop in the activation of GPCR and FXR in enteroendocrine cells to sustain the production of GI hormones vital for energy homeostasis, which include GLP-1, GIP, PYY, and CCK. All these peptides have both peripheral and central effects on modifying the host metabolism and regulation of appetite directly through the vagus nerve or indirectly via immunoneuroendocrine mechanisms. Centrally, the aberrant hormonal signals transmitted from the gut to the hypothalamus in the brain result in aberrant eating behavior and metabolic control. While peripherally, the influx of LPS, pro-inflammatory cytokines, and bacterial DNA, along with inadequate SCFAs, Bas, and GI peptides in the circulation further exacerbates insulin signaling and metabolic imbalance. Altogether, these central and peripheral abnormalities in metabolic control eventually lead to hyperglycemia. 5-HT, 5-hydroxytryptamine; BA, BA; CCK, cholecystokinin; FXR, Farnesoid X receptor; GABA, γ-aminobutyric acid; GI, gastrointestinal; GIP, glucose-dependent insulinotropic polypeptide; GLP-1, glucagon-like peptide 1; GPCR, G-protein coupled receptor; LPS, lipopolysaccharide; PYY, peptide YY; SCFA, short-chain fatty acid; TLR4, Toll-like receptor 4.

However, the detrimental alterations in the gut microbiome disrupt the normal functioning of ENS and VN and cause aberrance in regulating metabolic control (126) (**Figure 7**). In normal conditions, SCFAs increase intestinal gluconeogenesis and improve peripheral glucose production and insulin sensitivity through a complex intestine-brain-neural circuit (59). SCFAs also improve short-term satiety and reduce weight gain (58, 146). Likewise, BAs are also associated with the regulation of body weight, food intake, and glucose homeostasis (147, 148). Different bariatric surgery and animal models owe more credit in highlighting the role of BAs and microbiome-gut-brain axis in metabolic control (149), as all of these techniques are designed to reverse the metabolic disorders by surgically changing the anatomical and physiological nature of the GI tract, which is bound to induce alterations in intestinal microbiome (150).

In sum, there are many similarities between obesity and T2DM in genetic elements playing essential roles in their pathogenesis, which can be further affected and amplified by environmental factors. Internally, following the gradual reshaping of the microenvironment by the alterations in the metabolic state, the local aggregation of detrimental stress on pancreatic islets ends up with the deterioration of β-cell function or loss of the β-cell population in T2DM. Meanwhile, in addition to the intrinsic and profound impact of genetics on the microbiome, the remodeling of the intestinal microbiome by the over-eating dietary habit is the reason and the consequence of metabolic disorders. What is vital for energic homeostasis, the regulation of nutritional absorption by the intestines becomes chaotic in obesity and T2DM, with the disrupted gut-brain interactions fueling excessive energy consumption and weakening the restriction of energy intake, which not only directly promotes the occurrence of obesity and T2DM, but also aggravates metabolic abnormalities and concurrent complications via dysfunction of neuronal circuits dysregulation of various hormones.

## 4 The mutual effect of available treatment for obesity and T2DM

T2DM is a slowly progressing metabolic disease closely related to obesity. Hence, obesity management ameliorates or even remits the T2DM in many patients. Likewise, since nearly 2/3 of the patients with T2DM have a weight problem (151), some anti-diabetic treatments (such as oral medications) can also reduce body weight. In general, treatments effective for both obesity and T2DM include lifestyle interventions (such as dietary modification, physical activity, and behavioral therapies), pharmacotherapy, medical devices, and bariatric surgery.

### 4.1 Lifestyle interventions

Given their inexpensiveness and minimal side effects (28, 152, 153), lifestyle interventions are always the first option, or more precisely, the cornerstones for the management of obesity and T2DM (1, 154). What is the core of lifestyle interventions, behavioral strategies include a self-monitoring of body weight, blood glucose, diet, and physical activity, behavioral contracts and goal setting (1), which helps patients stick to a healthy lifestyle, eating habits, and the frequency and intensity of physical activity that contribute to weight loss and glycemic control. An increased energy expenditure based on calorie restriction (CR) is the primary driver of weight loss, helping patients to reduce body weight and lower blood glucose. In this context, lifestyle interventions include the prescription of a low-calorie diet, increased physical activity, and the development of behavioral strategies that enhance adherence to dietary and physical activity recommendations (155). In principle, all patients should be encouraged to gradually increase their physical activity while cutting calorie intake if they are physically capable. However, these interventions must be personalized following a complete evaluation of the patient’s condition and be adjusted according to individualized feedback. Most importantly, they require constant adaptation on a life-long basis regardless of whether other treatments are involved. Other emerging weight reduction methods, such as Internet-based weight-loss treatment and weight-loss apps for smartphones or other portable devices, are also gaining popularity. However, their efficacy is uncertain as these interventions are relatively passive and lack intensity (155).

### 4.2 Dietary modification and physical activity

Dietary modification (medical nutrition therapy) and physical activity are two primary and significant lifestyle interventions. Although there is no optimal diet or physical activity regimen for every patient, optional and professional guidelines have recommended various appropriate dietary patterns and multiple types of physical activities that facilitate energy restriction, induce weight loss, and improve glycemic status (152, 156).

Diets good for weight loss and glycemic control are characterized by low energy density and high dietary fiber. Specifically, a diet that includes high-quality lean protein, vegetables, fruits, some healthy fats (such as nuts and avocados), and a limited intake of refined carbohydrates would be optimal for most patients (1), which makes a vegetarian diet and a ketogenic diet (KD) preferable. KD was suggested to improve systemic metabolic signaling by reducing blood glucose and insulin while ameliorating insulin sensitivity, reducing body weight, and preventing cancer in rodents and humans (157, 158). In addition, convenient and calorie-traceable meal replacements diets and more tolerable and acceptable intermittent fasting are getting increasingly popular nowadays as an alternative to the traditional methods of CR. However, no matter which diet pattern is chosen, the ongoing adherence to the diet weighs more than the dietary composition itself in weight loss and glycemic control (1). Meanwhile, regular physical activity has many delectable health benefits, which will further improve CR results if combined with other approaches (1).

Undoubtedly, patients adhering to these diets and exercise plans will benefit from the improved physical condition and decreased risk of many other diseases. Nevertheless, apart from clearly indicated pharmacotherapy, with lifestyle interventions alone, many patients may “plateau” or find it hard to achieve personal targets consistently over a more extended period, or even worse, have a “rebound” in body weight and blood glucose. Under these circumstances, pharmacotherapies usually start when these “vulnerable” improvements depending on persistence and a life-long adaptation stop working.

### 4.3 Pharmacotherapy

Considering the limited efficacy of lifestyle interventions, other therapeutic approaches are needed to maintain or strengthen obesity and T2DM management effectively. Technically, both weight-loss and glucose-lowering medications must be taken for a long time unless they are intolerable, or cessation is highly indicated due to the safety concerns related to any possible side-effect. Since the patients’ diets and physical activity will not be affected by the drugs, it is still important to advise patients to stick to the concomitant lifestyle interventions.

Following the withdrawal of Locaserin by the Food and Drug Administration (FDA) in 2020 due to the concern regarding the increased risk of cancer (159), Orlistat, Phentermine-topiramate (PHEN/TPM), Naltrexone-bupropion (NB), and GLP-1 receptor agonists (GLP-1RAs) are currently available for obesity management, a systematic review and network meta-analysis comprising 143 randomized controlled trials (RCTs) provided solid data on the comparison of their efficacy and safety (160), and these anti-obesity medications have also been suggested to be effective in lowering blood glucose to some extent (152). Meanwhile, most anti-diabetic drugs can also impact body weight. For example, sodium-glucose cotransporter-2 inhibitors (SGLT-2is) can help lose weight, while insulin, one of the commonly used drugs, increases body weight (152). The mutual effects of available pharmacotherapy for obesity and DM are summarized in **Table 1**.

**Table 1:** The mutual effects of weight-lowering drugs and anti-diabetic drugs on managing obesity and DM.

Treatment	Mechanism of action (Not exhaustive)	Efficacy in weight loss/glycemic control	Advantages	Disadvantages	Impact on body weight/glycemic traits	Refs
Weight-lowering drugs						
Orlistat	① Lipase inhibitor ② Fat malabsorption	Weight loss compared with placebo (%, same below): -6.1%/-10.2%	① Certain efficacy ② Convenient	① High rates of side effects ② Limited durability ③ Expensive	① Efficacy of weight loss (compared with lifestyle modification alone): Phentermine-Topiramate>GLP-1RAs>Naltrexone-Bupropion> Orlistat ② Adverse events: Naltrexone-Bupropion>Phentermine-Topiramate>GLP-1RAs>Orlistat ③ Anti-diabetic effect: Intermediate	(28, 152, 155, 160, 161)
Phentermine-topiramate (PHEN/TPM)	① Phentermine: norepinephrine-releasing agent ② Topiramate: GABA receptor modulation ③ Appetite↓	-1.2%/-7.8% to 9.3% (dose- dependent)				
Naltrexone-bupropion (NB)	① Naltrexone: opioid antagonist ② Bupropion: dopamine and norepinephrine reuptake inhibitor ③ Food intake↓	-1.3%/-5.0% to -6.1% (dose- dependent)				
GLP-1 receptor agonists (GLP-1RAs)	① Liraglutide: obesity (3.0 mg, once daily)/T2DM (1.8 mg) ② Semaglutide (2.4 mg, once weekly) ③ Delayed gastric emptying	Liraglutide: -2.6%/-8% Semaglutide: -2.4%/-14.9%				
Anti-diabetic drugs						
Biguanides (Metformin, MET)	① Hepatic glucose output↓ ② Peripheral tissue sensitivity↑ ③ GLP-1 secretion↑	High	① Validated efficacy ② No hypoglycemia ③ Inexpensive ④ Only drug available for patients who are 18 years or younger	① GI symptoms and V_B12_ deficiency ② Dose adjustment/avoidance for renal disease ③ Lactic acidosis (rare)	↓	(151, 152, 162–164)
Sulfonylureas	Insulin secretion↑	High	① Validated efficacy ② Lowered microvascular risk ③ Inexpensive	① Hypoglycemia ② Uncertain cardiovascular safety ③ Dose adjustment/avoidance for renal disease ④ High rate of secondary failure	↑	
Thiazolidinediones (PPAR-γ agonists or TZDs)	Insulin sensitivity in target organs↑	High	① Low risk of hypoglycemia ② Long durability ③ Lipidemia↓ ④ Cardiovascular events↓ ⑤ Inexpensive	① Risk of edema and heart failure↑ ② Bone loss and bone fractures↑	↑	
Meglitinides (Glinides)	Insulin secretion↑	Intermediate-high	① Postprandial glucose fluctuation↓ ② Flexible dosage ③ Safe in advanced renal disease with appropriate dosing ④ Inexpensive	① Hypoglycemia ② Uncertain cardiovascular safety ③ Frequent dosing adjustment	↑	
GLP-1RAs	① Insulin secretion↑ ② Glucagon secretion↓ ③ Hepatic glucose output↓ ④ Delayed gastric emptying ⑤ Satiety↑	Intermediate-very high (depend on drug)	① No hypoglycemia as monotherapy ② Excellent postprandial glucose control ③ Cardiovascular risk↓	① GI side effects ② Heart rate↑ ③ Dose adjustment/avoidance in renal disease ④ Multiple complications ⑤ Very expensive	↓	
Dipeptidyl peptidase-4 inhibitors (DDP-4is)	① Insulin secretion↑ ② Glucagon secretion↓	Intermediate	① No hypoglycemia ② Satisfying tolerance	① Potential risk of urticaria/angioedema ② Hospitalization because of heart failure↑ (certain drug) ③ Dose adjustment/avoidance for renal disease	→	
Sodium glucose cotransporter-2 inhibitors (SGLT-2is)	Urinary glucose excretion↑	Intermediate-high (depend on glomerular filtration rate)	① No hypoglycemia ② Blood pressure↓ ③ Effective at all stages of T2DM with preserved glomerular function ④ Cardiovascular risk, heart failure, chronic kidney disease (certain drugs)↓	① Genital and urinary tract infections ② Polyuria ③ Volume depletion/hypotension/dizziness ④ LDL-C↑ ⑤ Creatinine↑ ⑥ Dose adjustment/avoidance for renal disease ⑦ Risk for amputation and fracture (canagliflozin) ↑ ⑧ Expensive	↓	
α-Glucosidase inhibitors	Slowed glucose absorption by delaying degradation of complex carbohydrates in the GI tract	Low-intermediate	① Low risk for hypoglycemia ② Postprandial glucose fluctuation↓ ③ Mechanism of action in non-systemic way ④ Cardiovascular safety ⑤ Inexpensive	① GI side effects ② Frequent dosing adjustment ③ Dose adjustment/avoidance for renal disease	↓	
Insulin	Insulin supplementation: ① Glucose disposal↑ ② Glucose production↓	Very high	① Universal response ② Satisfying efficacy	① Hypoglycemia ② Frequent dose adjustment ③ Expensive	↑	
Amylin analogues	① Glucagon secretion↓ ② Delayed gastric emptying ③ Satiety↑	Intermediate	Postprandial glucose fluctuation↓	① Hypoglycemia ② Frequent dosing adjustment ③ GI side effects ④ Very expensive	↓	
GIP/GLP1 dual agonist (Tirzepatide)	Synergistic incretin effect: ① Insulin secretion↑ ② Glucagon secretion↓ ③ Insulin biosynthesis↑ ④ β-cell proliferation↑ ⑤ β-cell apoptosis↓	① vs placebo: -17.71 mmol/mol (-1.62%) to -22.35 mmol/mol (-2.06%) ② vs GLP-1 RAs: -3.22 mmol/mol (-0.29%) to -10.06 mmol/mol (-0.92%) ③ vs basal insulin regimens: -7.66 mmol/mol (-0.70%) to -12.02 mmol/mol (-1.09%)	① Superior effect of glycemia control compared with placebo, GLP1RAs, and basal insulin ② Does not increase the odds of hypoglycemia	GI adverse events (mainly at high dose) such as nausea, vomiting, and diarrhea	Efficacy in body weight reduction: Tirzepatide>GLP-1RAs	

(T2) DM, (type 2) diabetes mellitus; GABA, γ-aminobutyric acid; GI, gastrointestinal; LDL-C, low-density lipoprotein cholesterol.

### 4.4 Medical devices

Due to the conservativeness and limited efficacy of lifestyle interventions and pharmacotherapy, along with the aggressiveness, irreversibility, and safety concerns of bariatric surgery, medical devices have been developed to fill in this gap as an eclectic solution for those who decline or are unfit for bariatric surgery (165). Currently, the available devices for the management of obesity include adjustable gastric band (AGB, see “Bariatric surgery”), intragastric balloons, electrical stimulation systems, gastric emptying systems, endoscopic endoluminal bypass liners (not FDA-approved), other types of devices or techniques (**Table 2**). The weight-loss efficacy of these devices is between pharmacotherapy and bariatric surgery, with a positive effect on T2DM and glycemic status. More importantly, these devices do not cause anatomical gut changes and are associated with faster recovery and fewer complications (165). Still, more data are needed concerning their long-term efficacy, safety, cost-effectiveness, and clinical use.

**Table 2:** The effects of medical devices on managing obesity and DM.

Treatment	Mechanism of action (Not exhaustive)	Efficacy in weight loss/glycemic control	Advantages	Disadvantages	Impact on body weight/glycemic traits	Refs
Intragastric balloons	① Gastric volume↓ ② Stimulation of afferent mechanosensitive receptors in the gastric wall ③ Delayed gastric emptying ④ Alterations in GI hormones	Total body weight loss (same below): 5-15%	① Globally used ② Easy placement and removal ③ Reversibility ④ Less invasive	① Temporary use only (6 months maximum) ② Modest weight reduction ③ GI side effects ④ Uncertain long-term safety ⑤ Weight regain	Improvement or remission of T2DM	(155, 165–174)
Electrical stimulation systems	① Gastric accommodation↓ ② Delayed gastric emptying ③ Satiety↑ ④ Calorie intake↓	9.2% at 1 year 8.0% at 2 years	Avoidance of permanent nerve damage	① Multiple side effects ② Relatively invasive and requires intraperitoneal violation (vagal nerve blockade)		
Gastric emptying systems	① Fastened gastric emptying ② Caloric intake↓	15-20%	① Long durability ② No increase in eating disorder incidence ③ Technically easy ④ Approved for higher BMI	① Device-specific risks: electrolyte abnormalities, nausea, and vomiting ② Gastrostomy tube requires maintenance ③ Potassium chloride supplementation and PPIs are commenced to reduce acid loss and potential potassium depletion due to aspiration of gastric contents		
Endoscopic endoluminal bypass liners (**NOT FDA-approved**)	Limitation of intestinal bypass component of bariatric procedure	15-20%	①Reversibility ② Improved glycemic control	① Multiple side effects ② Uncertain long-term safety ③ Fluoroscopic placement		
Other (Duodenal mucosal resurfacing)	① Absorption of nutrients↓ ② Enhanced cretin effect	Mean weight loss of 3.1kg at 3 months Not significant at 6 months	① No incision ② Restored insulin sensitivity ③ Promotes weight loss	① Modest weight loss ② Fluoroscopy needed ③ More data required		
Hydrogel particle	① Gastric volume↓ ② Satiety↑ ③ Calorie intake↓	5-15%	① Long durability ② No increase in eating disorder incidence ③ Convenient	① Uncertain long-term safety ② Modest weight loss ③ More data required ④ Expensive	Improved insulin sensitivity	

(T2) DM, (type 2) diabetes mellitus; BMI, body mass index.

### 4.5 Bariatric surgery

Bariatric surgery is the most effective treatment for both short- and long-term weight loss and glycemic control (10), and the safety of bariatric procedures has been greatly improved over the last two decades thanks to the increased experience and training of surgeons, use of laparoscopic and robotic approaches, improved perioperative and post-operative care and disciplines (175). Therefore, bariatric surgery has gained global popularity, with about half a million procedures performed annually (176). In addition, bariatric surgery has been included in many authoritative guidelines since it is a safe and effective treatment for severe obesity that results in long-term weight loss, improvement and remission of obesity-related comorbid conditions (particularly T2DM), metabolic syndrome, quality of life, and prolonged survival (177). Through complicated mechanisms beyond the magnitude of weight loss alone that includes improvements in incretin profiles, insulin secretion, insulin sensitivity, and so on, bariatric surgery achieves substantial, durable weight loss and glycemic control (28), which is more robust in long-term control of T2DM compared to conventional medication (178). The four most common bariatric procedures performed worldwide are sleeve gastrectomy (SG), Roux−en−Y gastric bypass (RYGB), adjustable gastric banding (AGB), and biliopancreatic diversion with duodenal switch (BPD-DS) (162, 177). These techniques induce significant weight loss and improvement or, very likely, the remission of T2DM, where SG and RYGB are the two dominant procedures worldwide, with the latter being superior in weight reduction and glycemic control but more complications and reoperation (10, 179). As for the AGB is rarely performed because of its lower weight loss, need for adjustment, and potential mechanical complications (1). Counting for less than 2%, BPD-DS is a combined and complex procedure that ranks the highest in weight loss, T2DM remission, and complications. Other widely endorsed miainstream techniques include Single-anastomosis duodenal ileostomy with sleeve gastrectomy (SADI-S) and One anastomosis gastric bypass (OAGB) (**Table 3**).

**Table 3:** The effects of bariatric surgery on managing obesity and DM.

Treatment	Mechanism of action (Not exhaustive)	Efficacy in weight loss/glycemic control	Advantages	Disadvantages	Impact on body weight/glycemic traits	Refs
Sleeve gastrectomy (SG)	① Gastric volume↓ ② Fastened gastric emptying ③ Food intake and calorie consumption↓ ④ GI hormones (ghrelin) ↓ ⑤ Appetite↓ ⑥ Satiety↑	1 year: 20-28% ≥6 Years: 22%	① Easier procedure Tends to avoid iron calcium and vitamin deficiencies ② Rapid and substantial weight loss ③ No foreign material implanted ④ Can be used as the initial procedure before RYGB or BPD–DS	① Risk of gastric leaks ② Late complications requiring conversion to RYGB ③ Weight regain due to dilated sleeve ④ Increased risk of postoperative GERD	① Popularity: SG>RYGB>AGB>BPD-DS ② Efficacy of weight loss and T2DM remission: BPD-DS>RYGB>SG>AGB ③ Complications: BPD-DS>RYGB>SG>AGB ④ Common mechanisms: Changes in hunger, satiety, energy balance, gastric pouch emptying rates, vagal signaling, GI hormone activity, circulating BAs, and the gut microbiome, Changes in inflammatory and adipokine profiles	(10, 28, 155, 162, 177, 180–182)
Roux-en-Y gastric bypass (RYGB)	① Food and calories consumption↓ ② Fat malabsorption ③ Calories and nutrients absorption↓ ④ Anti-incretin substances↓ ⑤ Incretin substance secretion↑ ⑥ Insulin sensitivity↑	1 year: 23-43% ≥6 Years: 25-28%	Notable long-term weight loss and glycemic control	① Complexity ② Long-term vitamin and/or mineral deficiencies ③ Longer hospital stay ④ Higher perioperative and late complications		
Adjustable gastric band (AGB)	① Satiety↑ ② Delayed gastric emptying	1 year: 14-30% ≥6 Years: 13-14%	① No surgical division of the stomach ② Shorter operative time ③ Reversibility and adjustability ④ Lower risk of vitamin and/or mineral malabsorption ⑤ Lower rate of death and perioperative complications	① Higher rate of reoperation for obstruction, band slippage or erosion ② Device vulnerability ③ Risk of band obstruction		
Biliopancreatic diversion with duodenal switch (BPD-DS)	① Food consumption↓ ② Absorption of protein, fat, nutrients, and vitamins↓ ③ Changes in GI hormones	<2 years: 48-64% ≥2 Years: 69-78%	① Highest weight loss and improvement in glucose metabolism ② Highest rate of remission of T2DM	① Complexity ② Higher complication rates and mortality ③ Potential deficiencies in proteins, vitamins, and minerals ④ Frequent follow-up required		
Single-anastomosis duodenal ileostomy with sleeve gastrectomy (SADI-S)	Similar to SG	21.5-41.2%, Without weight regain within 24 months after surgery	① Safe ② More simplified technique and less complications compared to BPD-DS ③ Shorter hospitalization ④ Strengthened efficacy in weight loss and glycemic control for patients with morbid obesity	① Complexity ② Higher complication rates ③ Potential deficiencies in in total serum proteins, folate, vitamin B12, calcium, and zinc	DM remission rate is up to nearly 75%	
One anastomosis gastric bypass (OAGB)	① Food intake and calorie consumption↓ ② Altered GI hormones↓ ③ Appetite↓ ④ Satiety↑ ⑤ Insulin sensitivity↑	EBMIL at a mean time of 3.2 ± 4.4 years: ① Revisional operations: 79.14 ± 14.8 ② Primary operations: 83.77 ± 13.41	① Safe ② Higher efficacy in weight loss and DM remission than RYGB and SG, respectively ③ Shorter operative time ④ Less complications	① Potential risk of bile reflux and stomal cancer ② Longer follow-ups and more data are required	① Weight reduction: AOGB≈RYGB>SG ② Average DM remission: 75.8% ± 12.2 at a mean time of 2.9 ± 3.4 years	

(T2) DM, (type 2) diabetes mellitus; EBMIL, excess body mass index loss.

Of course, bariatric surgery is not a “once and for all” or “one size fits all” measure to treat T2DM because some patients, especially those who are older, use insulin, regain weight, or have greater baseline waist circumference, longer diabetes duration, or poor preoperative glycemic control appear to be more likely to suffer from relapse of T2DM over time (183). But delightfully, the long-lasting effect of bariatric surgery can make recurrent T2DM less severe and easier to control with less intensive medication regimens (162).

To sum up, while lifestyle interventions, medical devices, and bariatric surgery are effective in treating both obesity and T2DM with the weight loss drugs have an intermediate impact on glycemic control, some anti-diabetic medicines such as insulin, sulfonylureas, TDZs, meglitinides have a negative weight-increasing effect, which may contribute to obesity-related comorbidities.

## Closing remarks

Unquestionably, consistent growth of the population with obesity and T2DM is to be witnessed regarding the epidemiological data demonstrated above. Still, the prevention and tackling of obesity provide the opportunity to lighten the future burden of T2DM. The intricate connections in the pathogenesis of obesity and T2DM primarily lie in the magnifying impacts of obesity on genetic susceptibility and environmental factors. In detail, the remodeled and reshaped metabolic microenvironment in obesity drastically dampens insulin signaling and favors the gradual elevation of blood glucose owing to the excessive detrimental accumulation of certain nutrients and metabolites, hyperactive low-grade inflammation, disrupted autophagic process, and energy imbalance resulting from dysregulated microbiome-gut-brain axis. All these changes are predominantly caused by the widespread ectopic expansion of adipose tissue fueling the systemic reprogramming of immunometabolism while locally toxicating the pancreas and diminishing the numbers of functional β-cells.

Given these connections, most treatments available for obesity and T2DM have a mutual effect on each other. Future efforts are warranted in the work and multi-disciplinary collaborations in monitoring the prevalence and revealing the biological nature of obesity and T2DM to improve the accuracy of the diagnosis and the efficacy of the treatments by exploring more advanced and optimized therapeutic methods while maximumly guaranteeing their availability and accessibility and minimizing their side-effects and complications to immensely benefit the patients. It is noteworthy that prevention is still the most economic and long-lasting solution for obesity and T2DM, and only by striving together and taking decisive and comprehensive action can the whole world make real progress in this task that we have been failing for decades.

## Author contributions

QX and RR conceptualized the article. RR, TL, and XZ prepared the initial manuscript. RR prepared the figures. JS prepared the table. YC, RX, and XY did the literature search and helped organize the manuscript’s contents. RR, TL, XZ, and QX edited and revised the manuscript. All authors contributed to the article and approved the submitted version.
